# Dishevelled2 promotes apoptosis and inhibits inflammatory cytokine secretion in rheumatoid arthritis fibroblast-like synoviocytes through crosstalk with the NF-κB pathway

**DOI:** 10.18632/oncotarget.15172

**Published:** 2017-02-07

**Authors:** Xing Zhen Liu, Jie Fan, Ke Qi, Shu Peng Liu, Wei Dong Xu, Ying Gao, Xiao Dan Gu, Jia Li, Chen Guang Bai, Ye Qing Shi, Lan Ling Zhang, Dong Bao Zhao

**Affiliations:** ^1^ Department of Rheumatology and Immunology, Changhai Hospital, The Second Military Medical University, Shanghai, China; ^2^ Army Convalescence Area, Hangzhou Sanatorium of People's Liberation Army, Hangzhou, China; ^3^ Department of Joint Surgery, Changhai Hospital, The Second Military Medical University, Shanghai, China; ^4^ Experimental Center, Changhai Hospital, The Second Military Medical University, Shanghai, China; ^5^ Department of Pathology, Changhai Hospital, The Second Military Medical University, Shanghai, China

**Keywords:** rheumatoid arthritis, synovial fibroblast, apoptosis, inflammation, dishevelled2, Immunology and Microbiology Section, Immune response, Immunity

## Abstract

Dishevelled (Dvl) not only links the canonical Wnt and non-canonical Wnt pathways but can also crosstalk with other pathways. As there is no systematic study to date on Dvl in rheumatoid arthritis (RA), we explored the impact of Dvl2 on proliferation and inflammatory cytokine secretion in RA fibroblast-like synoviocytes (FLSs). Expression of Dvl2 in RA synovial tissue and RA-FLSs was measured. Dvl2 was overexpressed in collagen-induced arthritis rats and human RA-FLSs,. the apoptosis and secretion of inflammatory cytokines were observed. Genetic changes and corresponding mechanisms caused by overexpressing Dvl2 in RA-FLSs were assessed. Dvl2 was found to be overexpressed in RA synovial tissue and RA-FLSs. Overexpression of Dvl2 increased apoptosis and inhibited inflammatory cytokine secretion by RA-FLSs *in vivo* and *in vitro*, and Dvl2 inhibited expression of anti-apoptotic and inflammatory genes. One possible mechanism is that Dvl2 decreases the nuclear translocation of P65 and inhibits its ability to bind to the promoters of NF-κB target genes. Our findings reveal an underappreciated role of Dvl2 in regulating inflammation and RA-FLS apoptosis and provide insight into crosstalk between the Wnt and nuclear factor-κB (NF-κB) pathways.

## INTRODUCTION

Rheumatoid arthritis (RA) is a systemic and chronic inflammatory disease characterized by severe synovial hyperplasia and inflammation. The production of inflammatory mediators and tumor-like growth of RA fibroblast-like synoviocytes (RA-FLSs) due to insufficient apoptosis are the pathological hallmarks of RA [1]. Several signaling pathways are involved in the pathogenesis of synovitis, with the nuclear factor-κB (NF-κB) pathway being the most important [2, 3]. Indeed, targeting the NF-κB pathway is an important therapeutic strategy for RA, and inhibiting NF-κB (with anti-tumor necrosis factor(TNF) drugs) has shown good safety and efficacy in the treatment of this disease [4, 5].

In addition to the NF-κB pathway, the Wnt pathway, which is involved in organogenesis, cell proliferation, and tumorigenesis [6, 7], has an important function in the activation of RA-FLSs [8]. The Wnt pathway can be subdivided into β-catenin-dependent (Wnt/β-catenin) and β-catenin-independent (Wnt-planar cell polarity (Wnt-PCP) and Wnt-calcium (Wnt-Ca^2+^)) pathways [9]. Activation of Wnt/β-catenin results in the translocation of β-catenin to the nucleus, followed by its binding to TCF/LEF transcription factors. In the Wnt-PCP pathway, Wnt stimulates the small G protein RHO and RHO-associated kinase (ROCK), resulting in Jun kinase (JNK) activation. In the Wnt/Ca^2+^ pathway, Wnt5a leads to the release of intracellular Ca^2+^, which causes activation of Ca^2+^/calmodulin-dependent protein kinase II (CaMKII) [10]. Interestingly, all three Wnt pathways converge at the Dishevelled (Dvl) protein, which was originally discovered in Drosophila nearly 60 years ago [11].

Dvl is an essential component of the Wnt/β-catenin, Wnt-PCP, and Wnt-Ca^2+^ pathways [12–14]. Three Dvl homologs (Dvl1-3) have been distinguished in vertebrates, and of which contain four conserved domains (DIX, PDZ, DEP, and Dsh) [15, 16]. Dvl primarily acts as a positive regulator of the Wnt pathway. Several studies have reported that overexpression or inhibition of any of the three Dvls leads to the corresponding activation or suppression of Lef/Tcf-sensitive transcription [17, 18]. Moreover, Jiang et al. [19] discovered that Dvl is an essential component of Wnt receptor degradation, indicating that Dvl is also essential for negatively regulating the Wnt pathway. In addition to its dual role in the Wnt pathway, Dvl also can interact with dozens of other proteins through its conserved domains [20], and it has been shown that Dvl participates in crosstalk with other signaling pathways, such as the Notch, BMP, and TGF-β pathways [21–23]. In this study, we examined Dvl2 expression in RA synovial tissue and RA-FLSs. We evaluated the impact of Dvl2 on synovial inflammation and apoptosis in RA as well as functional interaction between Dvl2 and the NF-κB pathway.

## RESULTS

### Expression of Dvl2 is higher in RA synovial tissue and RA-FLSs

**Figure 1:**
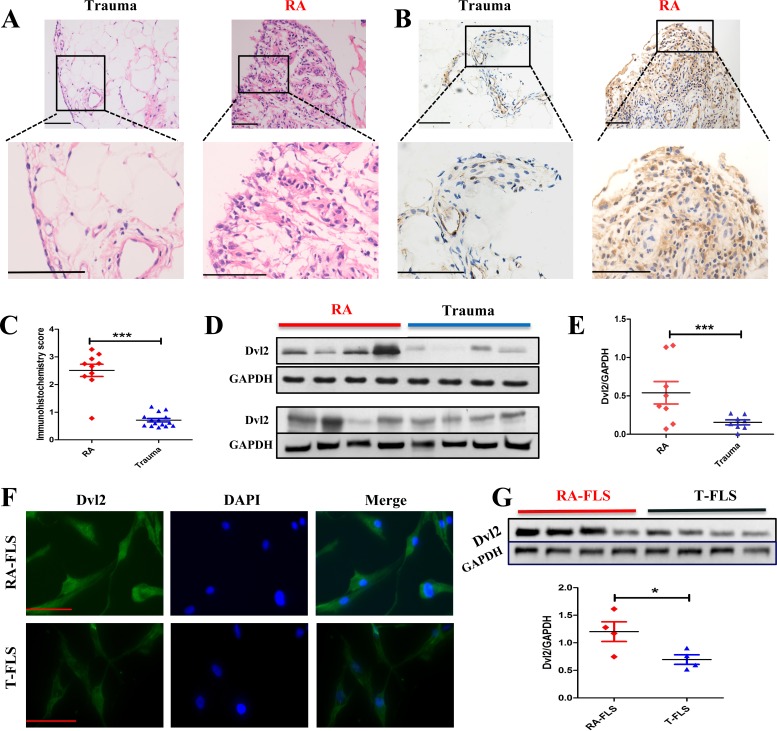
Expression of Dvl2 is higher in RA synovial tissue and RA-FLSs **A.** H&E staining was performed to identify synovial tissues from Trauma (*n* = 15) or RA (*n* = 10) groups. **B., C.** Synovial tissues from Trauma (*n* = 15) and RA (*n* = 10) patients were analyzed for Dvl2 expression using immunohistochemistry. Typical images and immunohistochemistry scores are shown. **D., E.** Synovial tissues from RA (*n* = 8) and Trauma (*n* = 8) patients were analyzed for Dvl2 expression by WB. **F.** The distribution of Dvl2 in primary human RA-FLSs and T-FLSs was observed by immunofluorescence. **G.** RA-FLSs (*n* = 4) and T-FLSs (*n* = 4) were analyzed for Dvl2 expression by WB. *p < 0.05, **p < 0.01, ***p < 0.001; Trauma, knee joint trauma; RA, rheumatoid arthritis; FLSs, fibroblast-like synoviocytes; WB, Western blotting. Scale bars: 100 μm.

A previous study demonstrated that β-catenin is upregulated in RA synovial tissue and RA-FLSs and that this contributes to stable activation of the Wnt/β-catenin pathway in these cells [24]. Accordingly, we sought to determine whether Dvl2 expression, similar to β-catenin, is also increased in RA synovial tissue. Synovial membranes were obtained from patients with knee joint trauma (the Trauma group) or RA, and hematoxylin-eosin (H&E) staining was performed to identify the synovial tissue (Figure 1A). The results of immunohistochemical analysis (Figure 1B, 1C) and western blotting (Figure 1D, 1E) showed significantly enhanced Dvl2 expression in the RA group compared with the Trauma group.

To identify isolated FLSs, levels of CD55 and vimentin expression were detected by flow cytometry, and the results are presented in Supplementary Figure 1. The intracellular distribution of Dvl2 in primary human RA-FLSs and FLSs isolated from Trauma patients (T-FLSs) was observed by immunofluorescence. Consistent with previous studies [25], Dvl2 was located in the cytoplasm and nucleus of FLSs (Figure 1F). Because the subcellular localization of Dvl may be crucial for local signaling events [15, 25], we also examined the nuclear localization of Dvl2. Immunohistochemical (Figure 1B, 1C) and immunofluorescence (Figure 1F) revealed a greater degree of Dvl2 nuclear localization in RA-FLSs than in T-FLSs.

Western blotting was performed to further compare Dvl2 expression in RA-FLSs and T-FLSs. The results showed significantly higher Dvl2 expression in RA-FLSs from four different RA patients compared to T-FLSs from four different Trauma group patients (Figure 1G).

### Overexpression of Dvl2 prevents joint destruction in the knee joint of CIA rats

**Figure 2:**
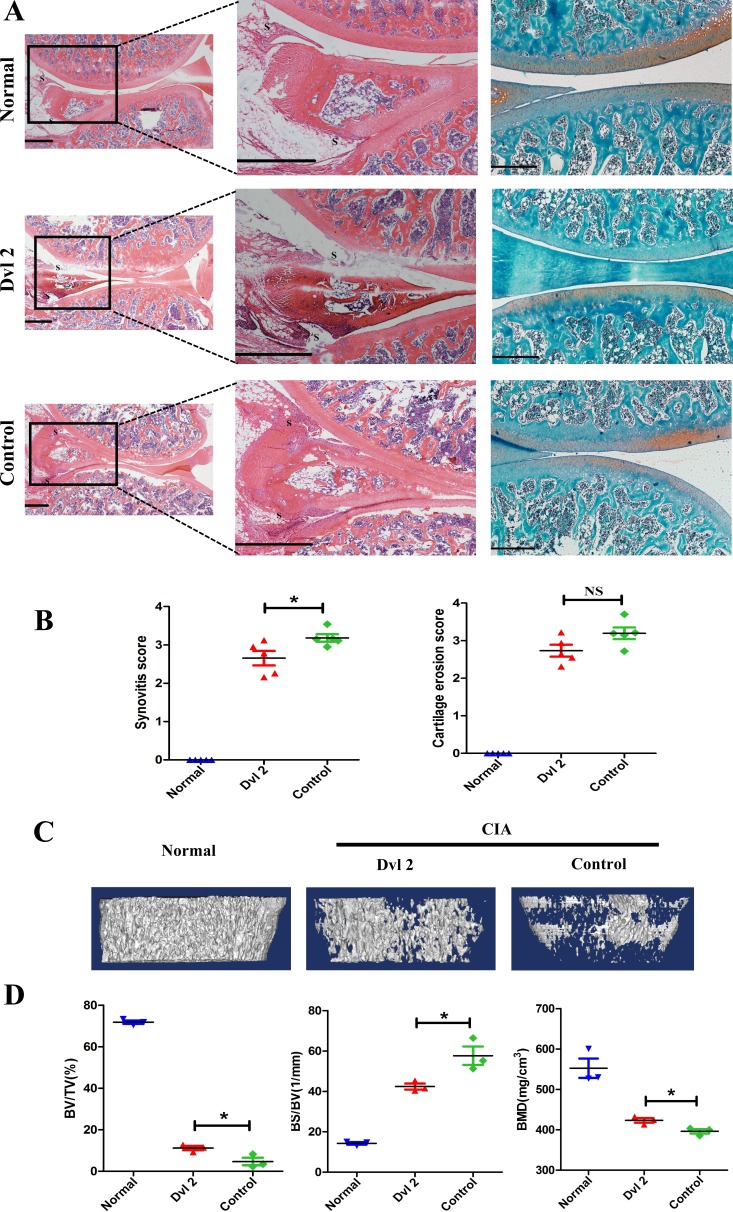
Overexpression of Dvl2 prevents joint destruction in the knee joint of CIA rats **A.** H&E and safranin O-fast green staining of knee joints (*n* = 5/group). **B.** Histopathological scores of synovial inflammation and cartilage destruction (*n* = 5/group). **C.** Micro-CT images of knee joints (*n* = 3/group). **D.** The measurement of bone volume/tissue volume (BV/TV), bone volume/total tissue volume (BS/BV), and bone mineral density (BMD) (*n* = 3/group). *p < 0.05, ^NS^*p* > 0.05; CIA, collagen-induced arthritis; Normal, healthy rats without CIA; Dvl2, the knee joints of CIA rats injected with lentivirus-Dvl2; Control, the knee joints of CIA rats injected with lentivirus-control. Scale bars: 500 μm.

Dvl2 is overexpressed in cancers and is defined as a positive regulator of tumor cell proliferation and motility. Although knockdown of Dvl2 (Dvl2-siRNA) is most commonly applied to inhibit the proliferation of tumor cells, we wanted to investigate the effects of overexpressing Dvl2 in RA. Therefore, instead of knocking down Dvl2, we overexpressed Dvl2 in the knee joints of collagen-induced arthritis (CIA) rats and evaluated knee joint destruction. Surprisingly, compared with the control group, a thinner synovial membrane and less cell infiltration were observed in the Dvl2 group rather than the aggravation of synovitis (Figure 2A, 2B). However, overexpression of Dvl2 did not decrease cartilage destruction (Figure 2A, 2B).

According to a micro-CT analysis of the knee joints, the degree of bone erosion in the Dvl2 group was milder than that in the control group (Figure 2C). The percentages of bone volume/tissue volume (BV/TV), bone volume/total tissue volume (BS/BV), and bone mineral density (BMD) in the Dvl2 group were improved compared to the control group (Figure 2D). These results suggest that overexpression of Dvl2 also exerted some protective effects on bone erosion in CIA rats.

### Dvl2 ameliorates arthritis in CIA rats by increasing synovial apoptosis and decreasing levels of inflammatory cytokines

**Figure 3:**
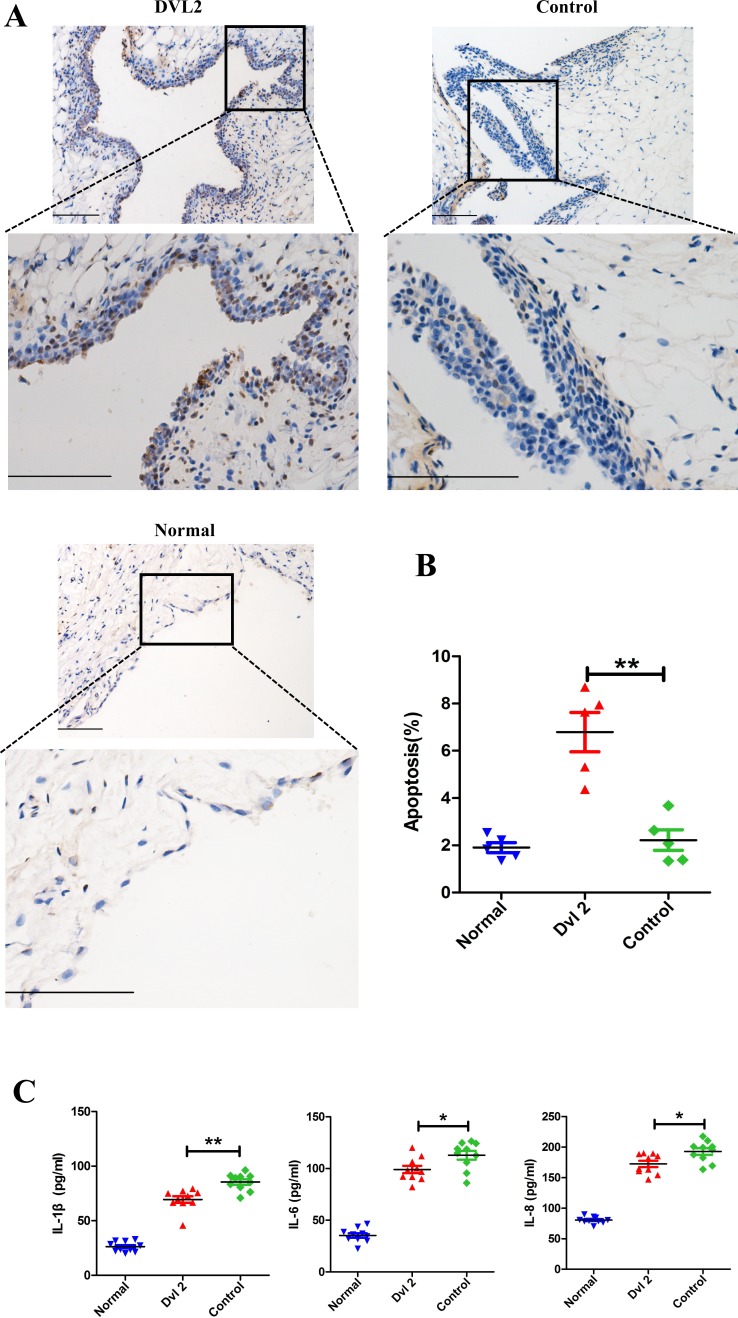
Dvl2 increases synovial apoptosis and reduces the secretion of inflammatory cytokines in the knee joints of CIA rats **A.** Synovial apoptosis in the knee joints was analyzed by TUNEL staining. **B.** Quantitative evaluation of TUNEL-positive synovial cells (*n* = 5/group). **C.** The level of intra-articular inflammatory cytokines was analyzed by ELISA (*n* = 10/group). *p < 0.05, **p < 0.01; CIA, collagen-induced arthritis; Normal, healthy rats without CIA; Dvl2, the knee joints of CIA rats injected with lentivirus-Dvl2; Control, the knee joints of CIA rats injected with lentivirus-control. Scale bars: 100 μm.

Histological analysis showed that Dvl2 reduced the thickness of the synovium in the knee joints of CIA rats. Therefore, we assessed whether this occurs through increased synovial apoptosis. TUNEL assay results showed that synovial apoptosis was significantly increased when Dvl2 was overexpressed in the knee joints of CIA rats (Figure 3A, 3B).

RA-FLSs can spontaneously secrete inflammatory cytokines, which play a crucial role in joint destruction in RA. Therefore, we detected changes in intra-articular inflammatory cytokines using enzyme-linked immunosorbent assay (ELISA) of the synovial fluid. We found that overexpression of Dvl2 significantly decreased IL-1β, IL-6, and IL-8 levels in the knee joints of CIA rats (Figure 3C).

### Overexpression of Dvl2 increases RA-FLS apoptosis by mitigating NF-κB-dependent expression of anti-apoptotic genes

**Figure 4:**
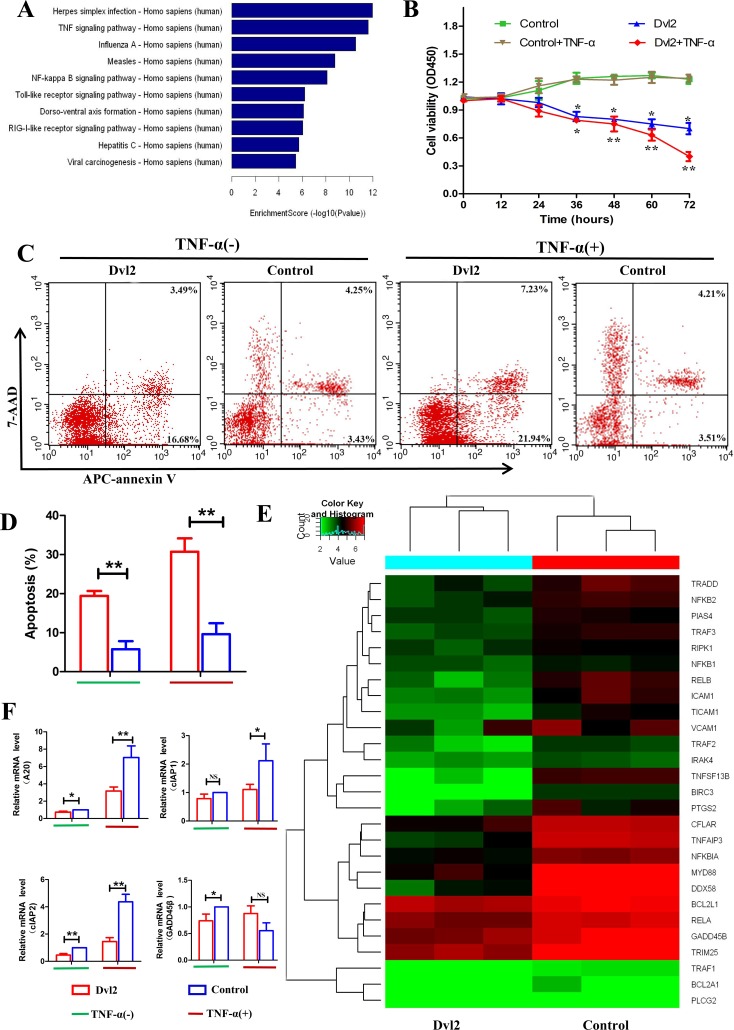
Overexpression of Dvl2 increases RA-FLS apoptosis by reducing expression of NF-κB-dependent anti-apoptotic genes **A.** The downregulated high-ranked pathways upon Dvl2 overexpression in RA-FLSs **B.** The viability of Dvl2-overexpressing RA-FLSs was assessed via CCK-8 assays at the indicated time points. **C.** RA-FLS apoptosis was analyzed by flow cytometry. **D.** The percentages of apoptotic cells are displayed in the histogram. **E.** Cluster diagram of the NF-κB signaling pathway when Dvl2 was overexpressed in RA-FLSs. Red indicates high relative expression, and green indicates low relative expression. **F.** The expression levels of NF-κB-dependent anti-apoptotic genes were validated by RT-PCR. *p < 0.05, **p < 0.01, ^NS^*p* > 0.05; Dvl2, RA-FLSs infected with lentivirus-Dvl2; Control, RA-FLSs infected with lentivirus-control.

Because overexpression of Dvl2 increased synovial apoptosis *in vivo*, RNA-seq was used to investigate the function of Dvl2 in RA-FLSs. We transferred lentivirus-Dvl2 into primary human RA-FLSs and observed GFP fluorescence using fluorescence microscopy to determine the infection efficiency (Supplementary Figure 2A). Western blotting was performed to verify overexpression of Dvl2 in RA-FLSs (Supplementary Figure 2B), and we then analyzed changes in mRNA levels using an Illumina HiSeq sequencer. The signaling pathway analysis revealed decreased expression of mRNAs involved in the TNF and NF-κB signaling pathways (Figure 4A), which are key in RA pathophysiology. The results suggest that Dvl2 has a close relationship with the NF-κB pathway, prompting us to explore the effects of Dvl2 on cell growth and cytokine release in RA-FLSs while using TNF-α to activate the NF-κB pathway.

A CCK-8 assay showed that overexpression of Dvl2 resulted in decreased RA-FLS viability, and this inhibition was more apparent after treatment with TNF-α (Figure 4B). Next, we analyzed apoptosis in Dvl2-overexpressing RA-FLSs using Annexin V-APC/7-AAD staining (Figure 4C, 4D) and found increased levels of apoptosis in Dvl2-overexpressing RA-FLSs compared to control-transfected RA-FLSs. After treatment with TNF-α, overexpression of Dvl2 led to a significant increase in RA-FLS apoptosis.

The RNA-seq results revealed 3193 altered mRNAs when Dvl2 was overexpressed, including 1570 upregulated mRNAs and 1623 downregulated mRNAs; of the latter, 27 are involved in the NF-κB signaling pathway (Figure 4E), including some classic NF-κB-dependent anti-apoptotic genes (e.g., A20, GADD45β, and cIAP2). Because cIAP1 and cIAP2 share the same domain, cIAP1 was also listed as the authentication object. The results of real-time polymerase chain reaction (RT-PCR) showed that overexpression of Dvl2 led to decreased mRNA levels of A20, GADD45β, and cIAP2; except for GADD45β, this effect became more pronounced upon stimulation with TNF-α. Although Dvl2 alone did not provoke to a significant decrease in cIAP1 mRNA levels, the inhibitory effect became apparent upon stimulation with TNF-α (Figure 4F).

### Overexpression of Dvl2 inhibits TNF-α-induced inflammatory cytokine secretion by RA-FLSs

**Figure 5:**
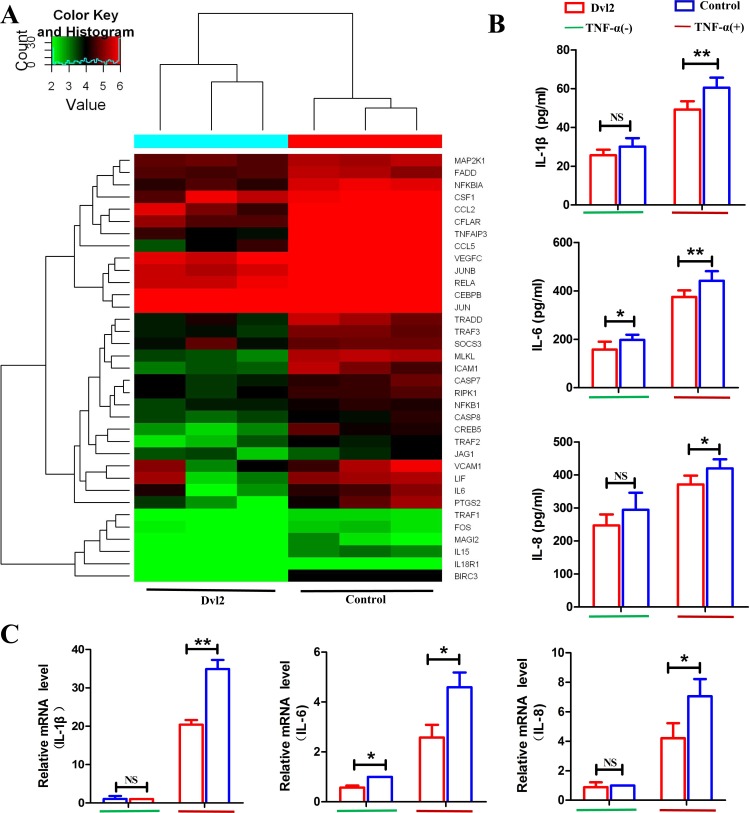
Overexpression of Dvl2 inhibits TNF-α-induced inflammatory cytokine secretion by RA-FLSs **A.** Cluster diagram of the TNF signaling pathway when Dvl2 was overexpressed in RA-FLSs. Red indicates high relative expression, and green indicates low relative expression. **B.** Cell supernatant levels of IL-1β, IL-6, and IL-8 in RA-FLSs were analyzed by ELISA. **C.** The mRNA expression levels of IL-1β, IL-6, and IL-8 were validated by RT-PCR. ^NS^*p* > 0.05, *p < 0.05, **p < 0.01; Dvl2, RA-FLSs infected with lentivirus-Dvl2; Control, RA-FLSs infected with lentivirus-control.

As we observed that Dvl2 can reduce the release of IL-1β, IL-6, and IL-8 *in vivo*, we next evaluated whether this effect is the result of significant apoptosis of RA-FLSs or a direct effect of Dvl2. Our RNA-seq results showed 35 downregulated genes involved in TNF signaling (Figure 5A), including IL-6, and we accordingly speculated that Dvl2 may directly decrease secretion of inflammatory cytokines by RA-FLSs. We performed ELISA analysis using cell supernatants and found that the cellular release of IL-6 was reduced by Dvl2 overexpression with and without TNF-α stimulation. Although Dvl2 alone did not reduce the release of IL-1β and IL-8, the inhibitory effects became apparent upon TNF-α stimulation (Figure 5B). Based on RT-PCR detection of IL-1β, IL-6, and IL-8 mRNA levels, Dvl2 alone reduced IL-6 expression, whereas IL-1β, IL-6, and IL-8 levels were only reduced upon TNF-α stimulation (Figure 5C). These results show that Dvl2 can directly inhibit inflammatory cytokine secretion by RA-FLSs *in vitro*, with the effect being more pronounced when the NF-κB pathway is activated by TNF-α.

### Dvl2 inhibits the NF-κB pathway by suppressing P65translocation and promoter binding

**Figure 6:**
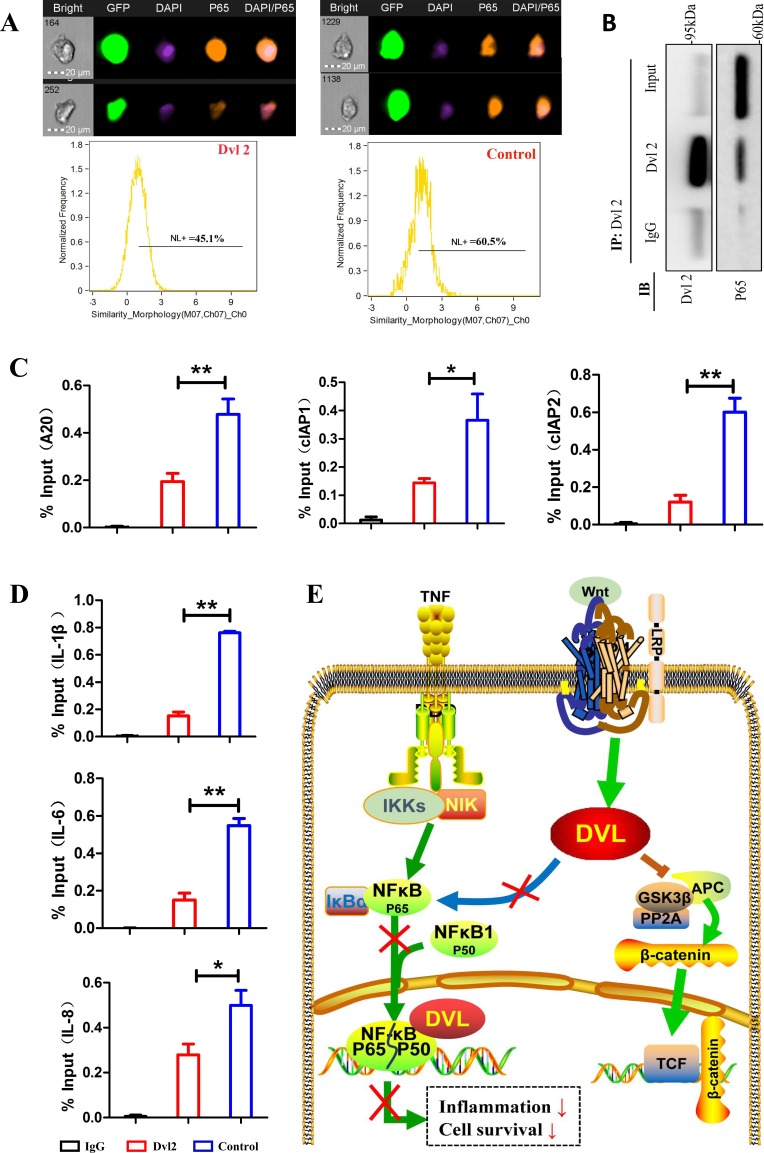
Dvl2 inhibits the NF-κB pathway by reducing the translocation and promoter binding of P65 **A.** Nuclear translocation of P65 was assessed using FlowSight. **B.** IP analysis shows that Dvl2 can interact with P65 in RA-FLSs. **C., D.** TNF-α-induced P65 binding to the promoters of anti-apoptotic and inflammatory genes was detected by a ChIP assay. **E.** The possible mechanisms by which Dvl2 inhibits the NF-κB pathway. *p < 0.05, **p < 0.01; Dvl2, RA-FLSs infected with lentivirus-Dvl2; Control, RA-FLSs infected with lentivirus-control.

To further investigate how Dvl2 reduces inflammatory cytokine and anti-apoptosis genes largely regulated by the NF-κB pathway, we focused on functional crosstalk between Dvl2 and the NF-κB pathway. Because nuclear translocation of P65 is an important prerequisite for activation of the NF-κB pathway, we first assessed this event using FlowSight. The results showed that overexpression of Dvl2 inhibited TNF-α-induced nuclear translocation of P65 (Figure 6A). Deng N et al. reported that Dvl can interact with P65 in HEK293T cells [26], and our immunoprecipitation (IP) analysis also showed interaction between Dvl2 and P65 in RA-FLSs (Figure 6B). A subsequent chromosome immunoprecipitation (ChIP) assay revealed that Dvl2 inhibited TNF-α-induced binding of P65 to the promoters of anti-apoptotic and inflammatory genes (Figure 6C, 6D).

Collectively, these results suggest that Dvl2 can decrease the expression of inflammatory cytokine and anti-apoptosis genes by inhibiting the NF-κB pathway in human primary RA-FLSs (Figure 6E).

## DISCUSSION

Although Dvl2 overexpression has been shown to contribute to cell survival and proliferation [27, 28], our data support an underappreciated role of Dvl2 in promoting apoptosis and inhibiting the secretion of inflammatory cytokines in RA-FLSs. Furthermore, we provide evidence for the existence of crosstalk between Dvl2 and the NF-κB pathway and that Dvl2 can inhibit target anti-apoptotic and inflammatory genes in RA-FLSs.

All three Dvls are expressed in mouse F9 cells and human HEK 293 cells, and Dvl2 constitutes the majority of the Dvl pool (80%-90%) [17]. Although there may be functional overlap among the three Dvl genes, Dvl2 has an important function in skeletal development [29, 30]. Therefore, we chose to analyze Dvl2 in this study. Expression of β-catenin is enhanced in RA synovial tissue and RA-FLSs, which exhibit tumor-like biological characteristics [24]. Consistently, we observed overexpression of Dvl2 in RA synovial tissue and RA-FLSs, suggesting that Dvl2 may involved in activation of the Wnt/β-catenin pathway and synovial hyperplasia in RA.

Unexpectedly, when Dvl2 exhibited a protective effect when overexpressed rather than knocked down in the knee joints of CIA rats; overexpression of Dvl2 also alleviated synovitis and bone erosion in CIA rats. The hallmark of RA is reduced apoptosis of synovioblasts, and the increased synoviocyte apoptosis caused by Dvl2 might explain the beneficial effects of Dvl2 with regard to pathological and radiological findings. In addition to mitigating synovitis, Dvl2 may have a direct protective effect on osteogenesis via activation of the Wnt/β-catenin pathway [31]. Unlike its function in bone metabolism, Dvl2's involvement in the Wnt pathway in cartilage homeostasis and disease is more complicated [32]. Regardless, overexpression of Dvl2 in the knee joints of CIA rats did not ameliorate cartilage destruction in the present study.

RA-FLSs can spontaneously secrete inflammatory cytokines, which play a critical role in the pathogenesis of RA [33]. These inflammatory cytokines are both the trigger and the target of the NF-κB pathway [34], and the increase in synoviocyte apoptosis caused by Dvl2 overexpression can lead to decreased secretion of inflammatory cytokines. In addition, RT-PCR results showed that Dvl2 indeed reduced the mRNA levels of IL-1β, IL-6, and IL-8 in RA-FLSs. Another interesting finding was that Dvl2 overexpression alone in RA-FLSs reduced the mRNA levels IL-6, whereas IL-1β, IL-6, and IL-8 were only significantly suppressed upon stimulation with TNF-α. This suggests that Dvl2 is active within the context of inflammation and can block positive feedback of inflammatory cytokines to the NF-κB pathway in RA-FLSs.

A20 and GADD45β are strong inhibitors of apoptosis and target genes of NF-κB [35, 36]. In the current study, the inhibitory effect of Dvl2 on A20, but not on GADD45β, became more pronounced upon TNF-α stimulation of RA-FLSs. This interesting finding may be related to functional difference regarding GADD45β, whereby GADD45β has a pro-apoptotic function in some cell types and diseases via the p38α-p53-Gadd45β axis [37]. The anti-apoptotic genes cIAP1 and cIAP2 are IAP family members induced by the NF-κB pathway [38, 39]. In this study, we found that Dvl2 alone inhibited cIAP2 but not cIAP1 in RA-FLSs, though both were significantly inhibited upon TNF-α stimulation; the reason for this difference may be because cIAP1 and cIAP2 vary with regard to expression and function [40].

Some researchers have reported that Dvl overexpression in Drosophila or COS-1 cells can lead to cell death [41, 42]. One explanation for this is that Dvl can form a complex with RAC to activate JNK, resulting in apoptosis [43–45]. In addition to stimulating the JNK pathway, our data indicate that Dvl2 can promote apoptosis of RA-FLSs by inhibiting the NF-κB pathway. Our mRNA-seq results showed that Dvl2 overexpression in RA-FLSs suppressed the NF-κB pathway by decreasing the nuclear translocation of P65 and interacting with it to inhibit its binding to the promoters of NF-κB target genes (anti-apoptotic and inflammatory genes). Therefore, we demonstrate that Dvl2 can decrease inflammatory activation and apoptosis resistance by inhibiting the NF-κB pathway. Our research adds new evidence for functional crosstalk between the Wnt and NF-κB pathways, the two most important cell survival and inflammatory pathways [46–48].

Taken together, our results provide new insight into the novel function and mechanism of Dvl2 in RA-FLSs, which are implemented via inhibition of the NF-κB pathway rather than through the Wnt pathway. These findings provide more information about Wnt-associated genes, crosstalk between the Wnt and NF-κB pathways, and the exploration of potential therapeutic targets for RA. Nonetheless, many mechanisms remain to be elucidated in future studies.

## MATERIALS AND METHODS

### Patients and synovial tissue samples

Synovial tissues were obtained from 10 patients with RA who underwent total knee replacement surgery. All of the patients fulfilled the American College of Rheumatology (ACR) diagnostic criteria [49]. The synovial tissues of 15 patients with knee joint trauma (the Trauma group) used for normal controls were obtained during arthroscopic examination or treatment. All patients lacked a history of immune diseases or other arthropathies. This study was approved by the Ethics Committee of the Shanghai Changhai Hospital. The clinical characteristics of the included patients are shown in Supplementary Table 1.

### Isolation and culture of fibroblast-like synoviocytes (FLSs)

A synovial tissue adherence method was used to obtain RA-FLSs and FLSs from the Trauma group patients (T-FLSs). In brief, synovial tissues were washed 2-3 times with phosphate-buffered saline (PBS) containing penicillin-streptomycin solution (GIBCO) and then cut into small pieces. The small pieces of synovial tissue were moistened with fetal bovine serum (FBS) (ScienCell) and affixed to a Petri dish. After inversion for 3-4 h at 37°C/5% CO_2_ in a humidified atmosphere, 20 mL of synoviocyte medium (ScienCell) was added to the Petri dish. After one week, the FLSs were harvested, and the process was repeated. To maintain good biological function of the FLSs, primary cells were used after 3-4 passages. To identify isolated FLSs, the expression levels of CD55 and vimentin were detected by flow cytometry.

### Animal studies

Fifty SPF male Wistar rats (6-8 weeks old) were purchased from Shanghai Laboratory Animal Center (China) and maintained at 21°C under a 12 h light/dark cycle. The rats had free access to a standard pellet diet and water. Ten of the rats were maintained as the normal controls (the Normal group). The remaining 40 rats were subjected to induction of collagen-induced arthritis (CIA). The CIA rat model was prepared according to the described methods [50].

Clinical arthritis was observed within 2 weeks after the primary immunization (Supplementary Figure 3). Twenty typical arthritic rats were selected according to arthritic score and divided into 2 groups on day 20. Lentivirus-Dvl2 was injected into one knee joint of the rats in the Dvl2 group, and lentivirus-control was injected into one knee joint of the rats in the Control group. The rats were sacrificed on day 35. Before opening the knee joint capsule, lavage fluid of the articular cavity was collected and analyzed by ELISA. The whole knee joints of 5 rats in each group were used for histopathological analysis, and 3 of them were used for micro-computed tomography (CT) analysis. The synovial membranes of another five rats in each group were isolated and used for TUNEL analysis.

### Overexpression of Dvl2

Lentiviruses encoding human Dvl2 were constructed and produced by Obio Technology (Shanghai). To determine whether the lentivirus stably infected the knee joint, lentiviruses encoding luciferase were injected into the knee joints of rats, and in vivo bioluminescence imaging (Caliper Life Sciences) was applied for confirmation (Supplementary Figure 4A). The genetic similarity between human and rat Dvl2 is 96.2% (Supplementary Figure 4B). Lentiviruses encoding human Dvl2 were injected into the knee joints of rats, and the synovial membranes were isolated two weeks later. The Dvl2 protein levels in the knee joints of rats were detected by western blotting (Supplementary Figure 4C).

RA-FLSs were infected with lentiviruses encoding human Dvl2. After 72 h of infection, the strength of GFP fluorescence was observed by fluorescence microscopy to determine the infection efficiency, and western blotting was performed to verify overexpression of Dvl2. Lentiviruses without the Dvl2 sequence were transfected into RA-FLSs as a negative control.

### Histopathological observation

Synovial tissues were fixed in formalin and embedded in paraffin, and immunohistochemistry was performed as previously described [51]. Dvl2-positive cells were counted at 20×10 magnification, and the scores were evaluated (0 = 0-9%, 1 = 10-24%, 2 = 25-49%, 3 = 50-74%, and 4 = 75-100%). The TUNEL assay was performed as previously described [52]. Positive FLS density was calculated as the number of apoptotic cells per square millimeter of section.

Hind knee joints were fixed in 10% phosphate-buffered formalin for 24 h and decalcified in 14% EDTA-glycerol for 30 days at 37°C. Knee joints were embedded in paraffin. H&E and safranin O-fast green staining were performed to examine synovitis and cartilage destruction. Lesion severity was evaluated according to published criteria [53, 54].

### Immunofluorescence

To detect the intracellular distribution of Dvl2 in RA-FLSs and OA-FLSs, immunofluorescence was performed as described previously [55]. The stained cells were visualized using a fluorescence microscope (OLYMPUS).

### Micro-CT analysis

Micro-CT micrographs of the knee joints were obtained using a micro-CT system (Skyscan 1172) at 16-μm isotropic voxel size. After scanning, CTAn software (Bruker) was used for three-dimensional analysis.

### Measurement of inflammatory cytokines

Lavage fluid of articular cavity was collected and used for cytokine analysis of knee joints. Infected RA-FLSs were unstimulated or stimulated with 10 ng/mL TNF-α for 24 h, and the culture medium was collected and used to analyze cytokine release from RA-FLSs. Levels of IL-1β, IL-6, and IL-8 in the lavage fluid of the articular cavity and cell culture supernatants were measured using an ELISA kit (R&D Systems) according to the manufacturer's instructions.

### Western blot assay

Synovial tissue and FLSs were adequately lysed using lysis buffer, and the supernatants were collected after centrifugation. Quantitative protein samples were resolved by sodium dodecyl sulfate (SDS)-polyacrylamide gel electrophoresis and transferred to a polyvinylidene fluoride (PVDF) membrane. The membranes were incubated with appropriate antibodies and visualized using enhanced chemiluminescence. The relative intensities of protein bands were quantified by densitometry (Gel Logic 2200).

### Quantitative real-time PCR (RT-qPCR)

Infected RA-FLSs were stimulated or not with 10 ng/mL TNF-α for 12 h, and total RNA was extracted using TRIzol (Invitrogen) according to the manufacturer's instructions. cDNA synthesis and RT-PCR were performed per the protocols of PrimeScript RT reagent Kit and SYBR Green (Takara). The primer sequences are given in Supplementary Table 2. The 2^−^ΔΔ^Ct^ method was used for quantification of the relative mRNA expression levels.

### Sequencing analysis of mRNA

Lentivirus-Dvl2 and lentivirus-control were infected into primary human RA-FLSs from 3 RA patients. Total RNA extraction was performed using TRIzol (Invitrogen), and the nucleic acid concentration and purity were measured using a NanoDrop, a Qubit and an Agilent 2100 Bioanalyzer (Agilent Technologies). mRNA expression was assessed using an Illumina HiSeq sequencer, and 1.5-fold differential expression was considered to be upregulation or downregulation.

### Cell viability assay

Infected RA-FLSs were seeded in 96-well plates (4000 cells per well) and allowed to adhere. The infected RA-FLSs were stimulated or not with TNF (10 ng/mL). At 0, 24, 48, and 72 h, the cells were treated with CCK-8 (Dojindo) at 10 µL/well and incubated at 37°C for 4 h. The absorbance of each well was measured at 450 nm.

### Flow cytometry analysis

For CD55 detection, FLSs were harvested, washed twice with PBS, suspended in 100 μL PBS and incubated with anti-CD55 (Abcam) for 15 minutes at room temperature protected from light. For vimentin detection, FLSs were fixed for 30 minutes at room temperature, washed twice with transmembrane fluid, suspended in 100 μL transmembrane fluid and incubated with anti-vimentin (Abcam) at 4°C overnight.

Infected RA-FLSs were stimulated or not with 50 ng/mL TNF-α for 24 h, collected and washed in PBS. To assess apoptosis, the RA-FLSs were stained with Annexin V-APC/7-AAD (BD Biosciences) and examined using a FACSCalibur flow cytometer (BD Biosciences).

### FlowSight analysis

Infected RA-FLSs were stimulated with 10 ng/mL TNF-α for 30 minutes, fixed for 30 minutes at room temperature, and washed twice with transmembrane fluid. The cells were then incubated with anti-P65 (Abcam) at 4°C overnight and analyzed by Millipore-Amnis FlowSight (EMD Millipore-Amnis). Nuclear translocation of P65 was analyzed using Amnis IDEAS software (Millipore-Amnis; v.6.1).

### Immunoprecipitation (IP) and chromosome immunoprecipitation (ChIP)

Uninfected RA-FLSs were harvested and scraped into lysate buffer. IP assays were performed as described previously [56]. An anti-Dvl2 antibody (Cell Signaling Technology) or IgG (negative control) were conjugated to magnetic beads and incubated at 4°C for 10 h on a rotary shaker. Immunological complexes were eluted and boiled, and western blotting was performed.

Infected RA-FLSs were stimulated with 10 ng/mL TNF-α for 30 minutes and harvested. ChIP assays were performed as described previously [57] following the manufacturer's protocol (Millipore). DNA electrophoresis was used to examine the effect of sonication (Supplementary Figure 5). Protein-chromatin complexes were immunoprecipitated using an anti-P65 antibody (Cell Signaling Technology) or IgG. The chromatin was eluted, and the cross-links were reversed. The DNA was then purified and detected by real-time PCR using the primer sequences listed in Supplementary Table 3.

### Statistical analysis

Data are expressed as the mean ± SD. Statistical analysis was performed using GraphPad Prism (GraphPad Software) and SPSS 18.0 (SPSS Inc.). Parametric data were compared using a two-tailed Student's *t*-test, and p values of < 0.05 were considered significant (*p < 0.05, **p < 0.01, ***p < 0.001, ^NS^*p* > 0.05).

## SUPPLEMENTARY MATERIALS FIGURES AND TABLES


