# Supplementary material for: Dishevelled2 promotes apoptosis and inhibits inflammatory cytokine secretion in rheumatoid arthritis fibroblast-like synoviocytes through crosstalk with the NF-κB pathway
Source: Oncotarget. 2017 Feb 7;8(8):12649–63. doi: 10.18632/oncotarget.15172 (PMC5355042; doi:10.18632/oncotarget.15172)
Supplement: Supplementary file 1 [file oncotarget-08-12649-s001.pdf]

Dishevelled2 promotes apoptosis and inhibits inflammatory cytokine secretion in rheumatoid arthritis fibroblast-like synoviocytes through crosstalk with the NF-κB pathway

Supplementary Material

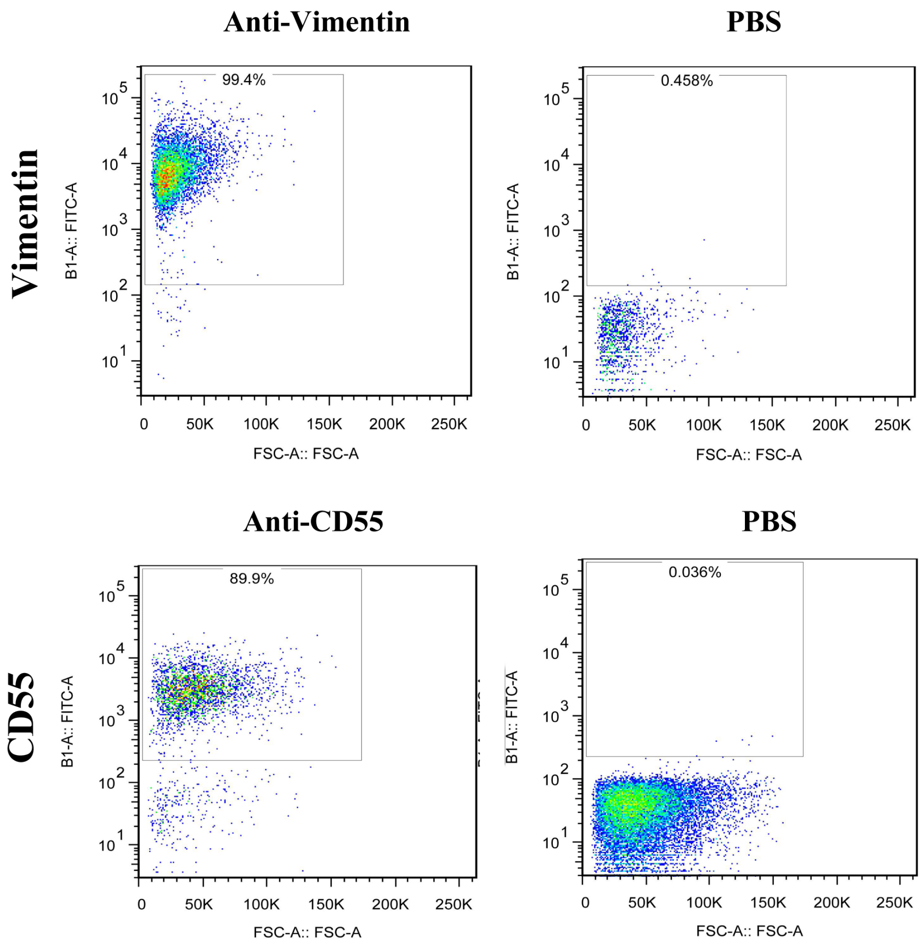

Figure S1 Fibroblast-like synoviocytes were identified by CD55 and vimentin.

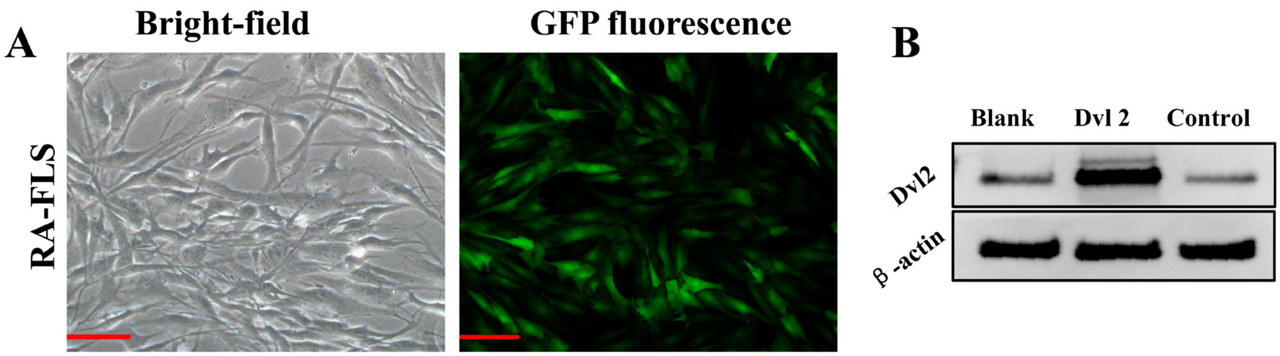

Figure S2 Over-expression of Dvl2 in RA-FLSs. (A) The strength of GFP fluorescence was observed by fluorescent microscopy after infecting lentiviruses encoding Dvl2 into RA-FLSs. (B) Over-expression of Dvl2 in RA-FLSs was detected by western blotting.

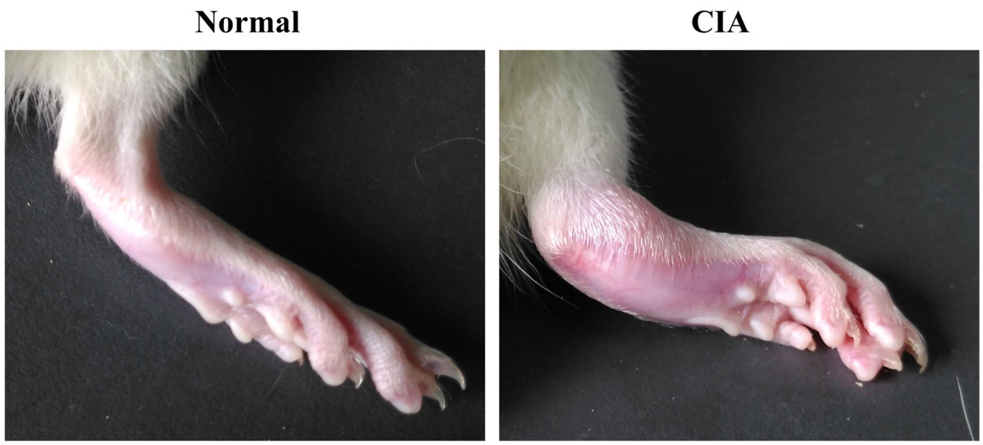

Figure S3 Clinical arthritis manifestations of red and swollen joints were observed in collagen-induced arthritis (CIA) rats.

A

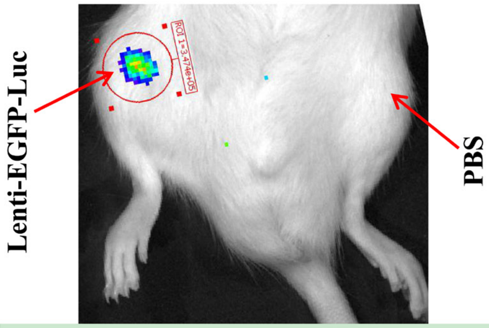

C

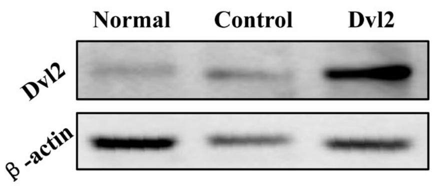

B

Gene similarity of Dvl2 between human and rat is 96.2%

```
Rat DVL2        MAGSSAGGGGVGETKVIYHLDEEETPYLVKIPVPAERITLGDFKSVLQRPAGAKYFFKSM
Human DVL2      MAGSSTGGGGVGETKVIYHLDEEETPYLVKIPVPAERITLGDFKSVLQRPAGAKYFFKSM
                *****.******************************************************

Rat DVL2        DQDFGVVKEEISDDSARLPCFNGRVVSWLVSSESPQPETAPPAHESRTELVPPPPPLPPL
Human DVL2      DQDFGVVKEEISDDNARLPCFNGRVVSWLVSSDNPQPEMAPPVHEPRAELAPPAPPLPPL
                ************** *****************.  **** *** ** *.** ** ******

Rat DVL2        PPERTSGIGDSRPPSFHPNVSSSHENLEPETETESVVSLRRDRPRRRDSSEHGAGGHRPG
Human DVL2      PPERTSGIGDSRPPSFHPNVSSSHENLEPETETESVVSLRRERPRRRDSSEHGAGGHRTG
                *****************************************.**************** *

Rat DVL2        VPSRLERHLAGYESSSTLMTSELESTSLGDSDEDDSMSRFSSSTEQSSASRLLKRHRRRR
Human DVL2      GPSRLERHLAGYESSSTLMTSELESTSLGDSDEEDTMSRFSSSTEQSSASRLLKRHRRRR
                 ********************************.*.************************

Rat DVL2        KQRPPRMERTSSFSSVTDSTMSLNIITVTLNMEKYNFLGISIVGQSNERGDGGIYIGSIM
Human DVL2      KQRPPRLERTSSFSSVTDSTMSLNIITVTLNMEKYNFLGISIVGQSNERGDGGIYIGSIM
                ******.*****************************************************

Rat DVL2        KGGAVAADGRIEPGDMLLQVNDMNFENMSNDDAVRVLRDIVHKPGPIVLTVAKCWDPSPQ
Human DVL2      KGGAVAADGRIEPGDMLLQVNDMNFENMSNDDAVRVLRDIVHKPGPIVLTVAKCWDPSPQ
                ************************************************************

Rat DVL2        AYFTLPRNEPIQPIDPAAWVSHSAALTGAFPAYPGSSSMSTITSGSSLPDGCEGRGLSVH
Human DVL2      AYFTLPRNEPIQPIDPAAWVSHSAALTGTFPAYPGSSSMSTITSGSSLPDGCEGRGLSVH
                ****************************.*******************************

Rat DVL2        MDMASVTKAMAAPESGLEVRDRMWLKITIPNAFLGSDVVDWLYHHVEGFPERREARKYAS
Human DVL2      TDMASVTKAMAAPESGLEVRDRMWLKITIPNAFLGSDVVDWLYHHVEGFPERREARKYAS
                 ***********************************************************

Rat DVL2        GLLKAGLIRHTVNKITFSEQCYYVFGDLSGGCESYLVNLSLNDNDGSSGASDQDTLAPLP
Human DVL2      GLLKAGLIRHTVNKITFSEQCYYVFGDLSGGCESYLVNLSLNDNDGSSGASDQDTLAPLP
                ************************************************************

Rat DVL2        GATPWPLLPTFSYQYPAPHPYSPQPPPYHELSSYSYGGGSASSQHSEGSRSSGSTRSDGG
Human DVL2      GATPWPLLPTFSYQYPAPHPYSPQPPPYHELSSYTYGGGSASSQHSEGSRSSGSTRSDGG
                **********************************.*************************

Rat DVL2        AGRTGRPEERAPESKSGSGSESELSSRGGSLRRGGEPGGTGDGGPPPSRGSAGGTPNLRA
Human DVL2      AGRTGRPEERAPESKSGSGSESEPSSRGGSLRRGGEASGTSDGGPPPSRGSTGGAPNLRA
                *********************** ************  ** **********.**.*****

Rat DVL2        LPGLHPYGPPPGMALPYNPMMVVMMPPPPPPVSTAVQPPGAPPVRDLGSVPPELTASRQS
Human DVL2      HPGLHPYGPPPGMALPYNPMMVVMMPPPPPPVPPAVQPPGAPPVRDLGSVPPELTASRQS
                 *******************************  **************************

Rat DVL2        FHMAMGNPSEFFVDVM
Human DVL2      FHMAMGNPSEFFVDVM
                ****************
```

Figure S4 Over-expression of Dvl2 in knee joint of rats. (A) Stable lentivirus infection in the knee joint of rats was confirmed by in vivo bioluminescence imaging. (B) Genetic similarity between human and rat Dvl2. (C) Over-expression of human Dvl2 in the synovial membrane of rats was detected by western blotting.

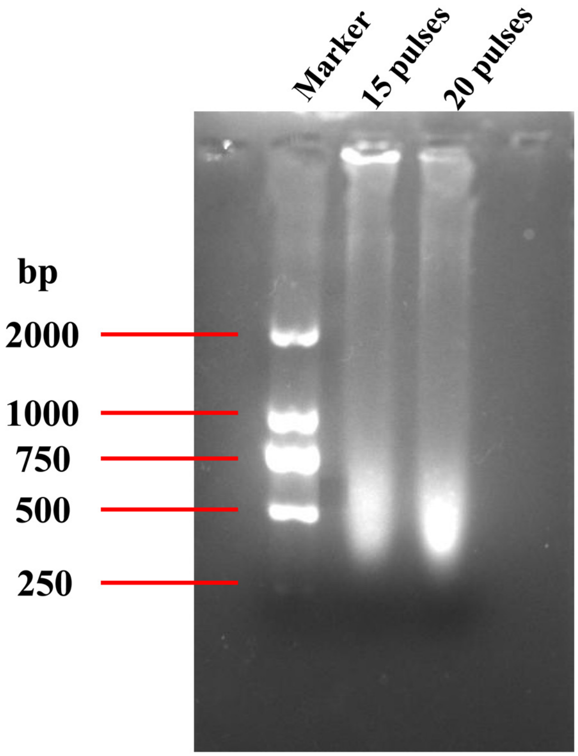

Figure S5 The effect of sonication detected by DNA electrophoresis.

Table S1 Clinical features of patients with knee joint trauma (Trauma group) and rheumatoid arthritis (RA) included in the study

Characteristic	RA	Trauma
Number	10	15
Age (years)	52(32-69)	29.7(23-32)
Male:female (n)	3/7	9/6
Disease duration (m)	121.2(60-240)	3.1(1-6)
ESR (mm/h)	49.4(7-90)	6.7(3-18)
CRP (mg/L)	62.8(2-114)	5.8(2.4-14)
RF+: n (%)	4(40%)	0

RA, rheumatoid arthritis; Trauma, knee joint trauma;

ESR, erythrocyte sedimentation rate; CRP, C-reactive protein; RF, rheumatoid factor;

Table S2 Primers for Real time RT-PCR

Gene	Forward primer(5′-3′)	Reverse primer(5′-3′)
IL-1β	CCAGCTACGAATCTCCGACC	CATGGCCACAACAACTGACG
IL-6	CAATAACCACCCCTGACCCAA	TCTGAGGTGCCCATGCTACA
IL-8	CATACTCCAAACCTTTCCACCCC	TCAGCCCTCTTCAAAAACTTCTCCA
A20	GCG TTC AGG ACA CAG ACT TG	GCA AAGCCCCGTTTCAACAA
cIAP1	GTTTCAGGTCTGTCACTGGAAG	TGGCATACTACCAGATGACCA
cIAP2	TCCTGGATAGTCTACTAACTGCC	GCTTCTTGCAGAGAGTTTCTGAA
GADD45β	TCACGCTCATCCAGTCCT	CGGCTTTCTTCGCAGTA
β-actin	GCACCACACCTTCTACAATGAG	ACAGCCTGGATAGCAACGT

Table S3 Primers for ChIP real time RT-PCR

Gene	Forward primer(5′-3′)	Reverse primer(5′-3′)
IL-1β	TCCCTCCCTTAGTGCCAACTATGT	ACAGTCTCCACAGTTCTGCCATGT
IL-6	ACCCTCACCCTCCAACAAAG	GCCTCAGACATCTCCAGTCC
IL-8	GGGCCATCAGTTGCAAATC	TTCCTTCCGGTGGTTTCTTC
A20	CAGCCCGACCCAGAGAGTCAC	CGGGCTCCAAGCTCGCTT
cIAP1	TGACTGGCAGGCAGAAATGA	TTTGCCCGTTGAATCCGAT
cIAP2	AAAGTGTATGGCGGATGGAG	GTGCACTGGTGCTTTCCTTT
